# CampaignView, a database of policy platforms and biographical narratives for congressional candidates

**DOI:** 10.1038/s41597-025-05491-x

**Published:** 2025-07-15

**Authors:** Rachel Porter, Colin R. Case, Sarah A. Treul

**Affiliations:** 1 University of Notre Dame, Department of Political Science, Notre Dame, 46556 US; 2 University of Iowa, Department of Political Science, Iowa City, 52242 US; 3 University of North Carolina at Chapel Hill, Department of Political Science, Chapel Hill, 27599 US

**Keywords:** Politics, Government

## Abstract

Thousands of candidates run for the U.S. Congress each election season, yet we lack systematic information on the vast majority of these contenders. Consequently, fundamental questions about polarization, agenda-setting, and representation remain unanswered. We introduce CampaignView, a database of campaign platforms and biographical narratives drawn from congressional campaign websites. Our corpus covers 5,228 candidates, representing 86.9% of major-party, ballot-eligible contenders who ran for the U.S. House of Representatives between 2018 and 2022. Our text data was collected in real-time during each election cycle, parsed into relevant units of aggregation, and manually annotated for topical coverage. In sum, our data includes 43,465 platform points and 5,114 biographical narratives. We provide auxiliary information on candidates and their electoral contexts to supplement our data. We host data for public dissemination at https://campaignview.org. Information is crucial to a well-functioning democracy; the open-access tools and data we produce have broad utility for journalists, advocacy groups, voters, and researchers seeking information on congressional campaigns.

## Background & Summary

Electoral campaigns play a crucial role in democratic governance as they help educate and inform the public about electoral decisions. Candidates use campaigns to present themselves and their policy positions to the voters, who, in turn, incorporate this information into their vote choice^1–3^. Importantly, the effects of candidates’ campaign messaging extend beyond an informational function. Candidates strategically tailor their self-presentation to sway how voters evaluate them^4–6^. When candidates emphasize certain policies, they influence voter perceptions about these issues’ importance^7^, perpetuate party brands^8^, and shape media attention by making clear the issues that “define” the election^9^. Candidates’ messaging tactics also have implications beyond elections. The policy priorities that candidates emphasize during campaigns predict their subsequent legislative priorities^10–12^. Thus, elections can provide insights into politicians’ future behaviors within Congress. For these reasons, data on candidates’ self-presentation and policy positions in congressional campaigns is crucial to evaluating key questions across multiple disciplines related to political representation, issue polarization, voter behavior, strategic communication, and policy agenda-setting.

The dynamics of two-party competition in modern congressional elections place increased importance on analyzing campaign messaging within the context of primary elections. Most congressional districts today strongly favor one party, leading to predictably partisan outcomes in general elections^13^. Consequently, primaries have emerged as the pivotal stage for meaningful electoral competition^14^. This shift in competition incentivizes candidates—who tailor their behavior to align with voter preferences^15^—to prioritize the interests of their primary electorate when shaping their campaign strategy^16^. Candidates may be particularly sensitive to strategic considerations regarding self-presentation and policy positioning in primaries because these factors play an outsized role in dictating vote choice in electoral contexts where partisanship is held constant^17,18^. Examining campaign strategies during the primary election may, therefore, be the best way to understand the factors shaping candidate behavior in modern elections.

Campaign websites are one of the most detailed and comprehensive sources for data on candidate position-taking and self-presentation. Nearly all congressional candidates today have a campaign website, and these sites frequently feature a biographical narrative and platform of policy positions^12^. Candidates and their teams invest significant effort into crafting their website messaging because these sites serve as an informational “hub” in modern campaigns^19,20^. In fact, more than a dozen states provide direct links to campaign websites in their official listings of ballot-eligible candidates. Research shows that rhetoric on campaign websites encapsulates a candidate’s broader messaging strategy, often reflecting the themes communicated across other platforms^21,22^. Moreover, unlike other social and online media, website content faces no explicit time or space restrictions, allowing candidates to fully elaborate on the messages most critical to their campaigns.

Currently, there is no open-source database of text from congressional candidate campaign websites. Instead, researchers have individually replicated the labor-intensive task of manually downloading, parsing, and labeling text from archived sites, often relying on sources like the Internet Archive’s Wayback Machine (https://web.archive.org/) or the Library of Congress (https://www.loc.gov/collections/united-states-elections-web-archive/about-this-collection/). However, this retrospective approach to data collection has limitations, as many candidates’ websites are either not archived or archived inconsistently throughout the election cycle. At best, reliable data from web archives is available for only about half of all primary election candidates^23–25^. Existing research that has collected website data in real-time has limited its scope to general election candidates^19^ or a single election cycle^26^ because of the significant time and effort required to collect these data.

This article introduces CampaignView, an open-source database of congressional candidate policy platforms and biographical narratives. Our database affords five key features. **First**, text from campaign websites is collected in real time a week before each state’s primary election, ensuring information from virtually every available campaign website is cataloged consistently. Our collection includes text data for 86.9% of the 6,016 major-party, ballot-eligible candidates who ran in primary elections for the U.S. House of Representatives between 2018 and 2022. **Second**, we clean and parse website text into relevant units of aggregation. Specifically, we parse campaign platform text at the policy level, storing each platform point as a separate document. This allows for the flexible aggregation and disaggregation of text based on researchers’ needs. **Third**, using human annotators, we hand-label every campaign platform point for its policy area, assigning each document a Major Policy Topic code. We provide guidance regarding how our topical codes map onto other coding schemas, namely the Policy Agendas Project’s policy agenda codes (https://www.comparativeagendas.net/project/us) and the Congressional Research Service’s policy area field values (https://www.congress.gov/help/field-values/action-codes). We anticipate that this mapping will be useful for researchers focusing their analyses on how policy-specific positioning translates into future legislative behavior. **Fourth**, to maximize the utility of our database, we supplement our text data with candidate- and election-specific information. We additionally include unique identifiers from other datasets to expedite cross-database merging. **Fifth**, we provide user-friendly access to these texts via an interactive data platform at https://campaignview.org. Users can query the interactive platform to filter text data by candidate name, political party, year, and congressional district. This online platform broadens the accessibility of our database to a general audience, including journalists, firms, voters, advocacy groups, teachers, and students.

In the remainder of this article, we describe our data collection and text processing procedure. We then discuss trends in campaign platform missingness and validate auxiliary data on candidates, elections, and campaign platform policy content. Finally, we provide a series of example use cases and highlight avenues for future research employing this text data from congressional candidates’ campaign websites.

## Methods

Our population of interest is all major-party, ballot-eligible candidates who ran for the U.S. House of Representatives between 2018 and 2022. In total, 6,016 candidates ran for the House across these election cycles. This section outlines how we constructed our database and proceeds as follows. First, we describe how we identified and parsed relevant text from congressional candidates’ campaign websites. Next, we lay out our procedure for labeling campaign platform topical content. Finally, we discuss the supplementary data we collected on congressional candidates and their electoral contexts, as well as our procedure for appending unique identifiers from supplemental datasets.

### Identifying & Parsing Campaign Platforms

Our procedure for identifying and parsing text data from candidate campaign websites begins several months before a given state’s primary. We start by identifying all major-party candidates running for the U.S. House of Representatives in that state. We produce this list of names after the state’s filing deadline passes to ensure no ballot-eligible candidates are missed. State filing deadlines usually fall two to three months before the primary election date. We refer to materials produced by the National Council on State Legislatures to identify state primary election dates (https://www.ncsl.org/elections-and-campaigns). To produce each state’s list of candidate names, we reference state elections and voting websites, usually hosted by a Secretary of State or State Board of Elections; filings from the Federal Election Commission (FEC) are also referenced to check for alternative candidate name spellings.

Once we finalize the list of candidate names for a given state, we conduct an initial search to identify the campaign website URL for each candidate running in that state. We identify URLs by following links from online repositories (e.g., https://politics1.com), visiting candidate social media pages, and querying search engines (e.g., search for “elise stefanik congress new york 21st district” on Google). After conducting this initial search, we adjourn data collection until the week before the state’s primary election date. At that time, we conduct a secondary search for campaign website URLs, specifically focusing our efforts on candidates for whom no URL was identified in our initial search.

**Fig. 1:**
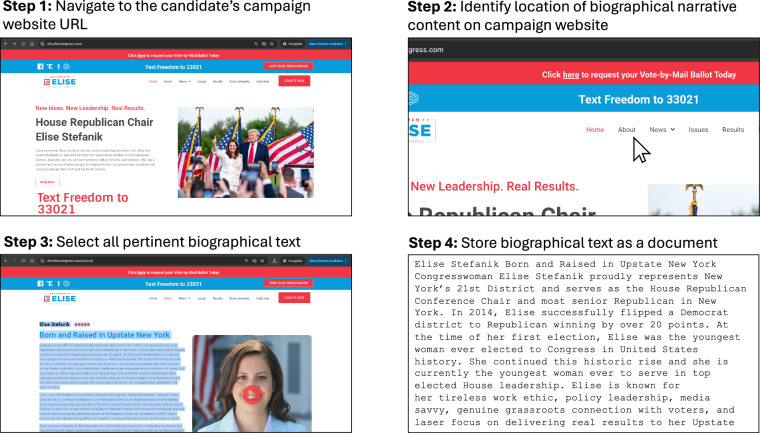
Illustration of the Procedure for Identifying and Storing Biographical Narratives from Campaign Websites. Steps depicted demonstrate the collection process conducted one week prior to each candidate’s primary election, using a representative example to illustrate each stage.

After ensuring the exhaustiveness of our search for campaign website URLs, we begin text data collection. We navigate to the URL associated with a given candidate to determine whether that site includes a biographical narrative. Biographical text is often hosted on a sub-page, accessible via a campaign website’s main menu with a title like “Meet the Candidate” or “About Me.” Occasionally, a biographical narrative is featured on the home page of a candidate’s campaign website. All biographical text on a candidate’s site is manually scraped and stored as a single document. Figure 1 depicts our collection procedure for identifying and storing biographical text.

**Fig. 2:**
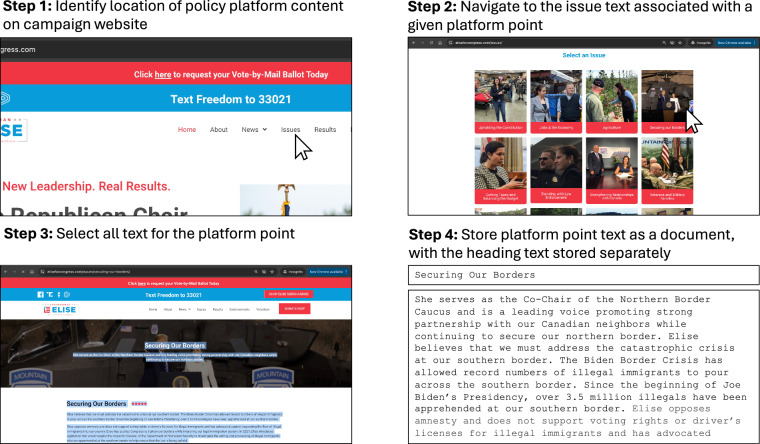
Illustration of the Procedure for Identifying and Storing Policy Platforms from Campaign Websites. Steps depicted demonstrate the collection process conducted one week prior to each candidate’s primary election, using a representative example to illustrate each stage.

We next determine whether that same candidate included a policy platform on their campaign website. This text is always hosted on a campaign website sub-page, accessible via a campaign website’s main menu with a title like “Issues,” “My Positions,” or “Where I Stand.” Policy platform text on campaign websites is organized as a series of platform points. All policy platform text on a candidate’s site is manually scraped and cataloged. Most often, these platform points are delineated by a subheading describing the associated text (e.g., “Ending Abortion,” “Reforming Immigration,” “Where I Stand on Climate Change”). We store each unique platform point as a document, including sub-heading text when available. Figure 2 depicts our collection procedure for identifying and parsing policy platform text on campaign websites. For a minority of cases, candidates list their platform points in a bulleted list, with each bullet featuring a distinct policy position. In these instances, we define a platform point as the text associated with each bulleted item. In rare cases, candidates only discuss their campaign platform policies in video format. We transcribe these videos and define each as a platform point.

We take several additional steps to maximize data coverage. First, we check the Internet Archive’s Wayback Machine—a platform that allows users to view archived versions of websites—for missed candidates. We restrict our search to the names of candidates for whom no data was collected during the primary election season (1,111 or 18.5% of all ballot-eligible candidates in our data). We collect only archived data timestamped within a month of a candidate’s primary election. Archived website data constitutes 7.8% of all candidates for whom we catalog campaign website text. Archived data represent significantly fewer candidates in more recent election years; archived websites were collected for 239 candidates in 2018, 101 candidates in 2020, and 67 candidates in 2022. Second, we expand the universe of websites scraped for incumbent members of Congress. A minority of incumbents either have no campaign website or their campaign website exclusively serves as a donation platform. In these instances, we identify the member’s official House.gov website and search this site for biographical and policy platform text. Text collected from House.gov websites is tagged as such in our data.

Using the data collection procedure described above, we successfully identified 5,300 congressional candidates as having a website—or 88.1% of all major-party, ballot-eligible candidates who ran for the U.S. House between 2018 and 2022. Of those candidates with a website, 85.0% included a policy platform, and 96.5% included a biographical narrative. A minority of candidates who had a website—a total of seventy-two across all election cyles, or 1.2%—did not present any biographical or policy information on that site. Validation of candidate inclusion, and trends in campaign website missingness are discussed in greater detail in ‘Technical Validation.’

### Labeling Campaign Platform Text for Topical Content

To provide greater insight into the contents of congressional candidates’ campaign platforms, we developed a comprehensive set of Major Policy Topic codes tailored specifically to electoral campaign content. Our team of human annotators manually labeled the entire corpus, consisting of over 40,000 platform points, assigning each of these documents to a single topical category. Annotators completed an in-person training session and practiced on a set of twenty example candidates, with their initial annotations checked for accuracy by a Principal Investigator (PI). Additionally, annotators were provided with a detailed instructional codebook containing examples to guide accurate topic assignment; this codebook is included in the Supplementary Materials. In cases where a platform point referenced multiple topics, annotators assigned the code corresponding to the majority of the text’s topical content. Validation of these hand-labeled documents is discussed in ‘Technical Validation.’

Tables 1 and 2 summarize the Major Policy Topic codes employed in hand-labeling. For each code, Table 1 provides illustrative examples of typical content, drawn from the annotation codebook provided in the Supplementary Materials. To provide further context, Table 2 presents the top words distinctly associated with each policy category compared to others, identified using a keyness analysis^27^. Details regarding applications for this coding schema are discussed in the ‘Usage Notes.’

**Table 1:** Example Contents of Major Policy Topics. Bulleted lists illustrate typical issues and policies categorized under each Major Policy Topic for classifying campaign platform content.

Major Policy Topic Code	Example Content
Agriculture	Trade protections for farmers
Civil Rights, Liberties, and Minority Issues	•Discrimination based on race, ethnicity, gender, sexuality, ability
	•Child abuse, domestic violence
	• Voting rights and participation
	•Women’s rights, LGBT rights, Native American rights
Crime	•War on Drugs, drug legalization
	•Reforming the criminal justice system
	•Reducing crime, improving public safety
	•Policing (e.g., Back the Blue, Defund the Police)
	•Gun rights, gun restrictions
Defense	•Domestic military spending; size/scope of the military
	•Supporting the troops; veterans’ welfare
Economics and Commerce	•State of the economy; local economies
	•Inflation, unemployment
	•Government regulation of commerce, tax policy
Education	•Higher education accessibility, tuition concerns
	•Parental rights, curriculum reform
	•Improving access to education for disadvantaged groups; access to pre-K
	•Discussions of homeschooling; charter, religious, or magnet schools
Energy and Environment	•Renewable energy, fossil fuels
	•Water, nature conservation, federal parks
	•Climate change, pollution
Government Operations	•Inter-branch relations, role/scope of the government
	•Government spending; debt, deficit
	•Bureaucracy expansion/contraction
	•Government reforms: term limits, size of Supreme Court, abolish the filibuster
Healthcare	•Affordable Care Act, Obamacare, Medicare for All, Medicaid
	•Prescription drug prices, drug affordability
	•Public health, disease prevention, vaccinations, addiction, mental health
Immigration	•Pathways to citizenship; DACA
	•Border security; Abolish ICE
International Affairs	•Discussions of approach to foreign policy (e.g., diplomacy, isolationism)
	•Discussions of specific country interactions (e.g., Russia, China, Israel)
	•Involvement in international organizations (e.g., NATO, United Nations)
	•International trade policies (e.g., NAFTA, bilateral agreements)
Social Welfare	•Affordable housing
	•Homelessness, poverty
	•Social Security
Transportation and Infrastructure	•Mass transportation
	•Roads, bridges, highways
	•Public works, infrastructure development

**Table 2:** Terms Associated with Major Policy Topics. Lists of terms are the top-ranked words distinctly associated with each Major Policy Topic, identified through a keyness analysis.

Major Policy Topic Code	Example Content
Agriculture	farmers, agriculture, farm, farms, farming, ranchers, agricultural, food, dairy, crops, farmer, producers, crop, rural, livestock, ag, organic, growers, agribusiness, corn, meat, usda, feed, markets, products
Civil Rights, Liberties, and Minority Issues	women, abortion, rights, lgbtq, equality, womens, discrimination, equal, reproductive, gender, life, sexual, pro-life, parenthood, planned, unborn, abortions, orientation, roe, marriage, lgbt, identity, transgender, wade, conception
Crime	gun, violence, guns, police, amendment, background, firearms, second, marijuana, weapons, checks, arms, criminal, crime, 2nd, enforcement, bear, justice, firearm, nra, safety, law, prison, shootings, officers
Defense	veterans, va, military, veteran, service, defense, served, affairs, civilian, men, troops, heroes, armed, uniform, sacrifices, brave, care, deserve, vets, ptsd, servicemembers, soldiers, sacrifice, benefits, duty
Economics and Commerce	tax, jobs, economy, small, businesses, wage, business, workers, taxes, minimum, economic, growth, wages, trade, job, class, unions, labor, manufacturing, code, income, middle, inflation, working, corporations
Education	education, students, schools, student, school, teachers, college, loan, public, children, colleges, debt, teacher, educational, parents, loans, learning, higher, tuition, universities, educators, teaching, pre-k, vocational, charter
Energy and Environment	energy, climate, clean, water, environment, change, environmental, renewable, oil, green, fossil, carbon, natural, gas, solar, emissions, air, wind, fuel, fuels, planet, pollution, sources, coal, lands
Government Operations	elections, election, voting, spending, limits, voter, campaign, government, money, budget, term, democracy, finance, vote, politics, candidates, voters, integrity, political, pacs, corruption, influence, gerrymandering, politicians, debt
Healthcare	healthcare, health, care, insurance, medicare, coverage, costs, affordable, prescription, obamacare, aca, premiums, pre-existing, medical, patients, medicaid, drug, cost, prices, single-payer, system, drugs, doctors, access, conditions
Immigration	immigration, border, immigrants, illegal, citizenship, borders, undocumented, wall, daca, immigrant, legal, dreamers, sanctuary, visa, asylum, illegally, ice, pathway, amnesty, aliens, deportation, southern, country, secure, patrol
International Affairs	israel, iran, foreign, peace, allies, nuclear, china, russia, sanctions, military, terrorism, east, international, diplomacy, korea, isis, israels, world, ukraine, security, war, terrorist, palestinians, threats, palestinian
Social Welfare	social, housing, seniors, security, retirement, medicare, homelessness, homeless, senior, benefits, rent, older, homes, disabilities, solvency, retirees, affordable, renters, privatize, retire, units, beneficiaries, income, rental, nycha
Transportation and Infrastructure	infrastructure, transportation, roads, transit, bridges, rail, highways, traffic, highway, projects, congestion, airports, crumbling, broadband, bus, bridge, freight, high-speed, airport, lanes, tunnel, repair, road, mta, commuters

### Matching Candidates with Auxiliary Data

During the text collection stage of database creation, we store candidate metadata found on campaign websites. The candidate information we collect includes history of elective experience (no elected experience, previously held public elected office), incumbency status (challenger, incumbent), and partisanship (Democrat, Republican). We also record the state and congressional district of a candidate’s electoral contest. After each election cycle, we collect information on each candidate’s primary election vote share, made available by State Election Offices. We also manually link each candidate with their identification number assigned by the Federal Election Commission. Procedures for collecting these auxiliary candidate data are outlined in our data collection codebook, provided in the Supplementary Materials.

## Data Records

The complete CampaignView database can be found on Harvard Dataverse^28^, distributed under the CC0 1.0 Universal license. The database comprises two datasets: the biographical narratives from candidate websites, and the campaign platforms from candidate websites. Each can be downloaded as a comma-separated file format (.csv, for access in programs such as Python), Stata data file format (.dta, for access in Stata), or R data file format (.rds, for access using the R programming language). The database is also publicly accessible and downloadable at campaignview.org. We describe each dataset in greater detail in the subsections below.

### Biographical Narratives

Each observation in this dataset is a biographical narrative for an individual candidate in a given year. An individual candidate is featured multiple times in the dataset if they ran across multiple election cycles. This dataset’s main feature of interest is the biographical narrative text taken from campaign websites. Text is complete and preserved of all formatting but has been cleaned of extraneous HTML source code. Additionally, characters incompatible with UTF-8 (e.g., ò and ą) were converted to plain text alternatives. Auxiliary information compiled during text collection about each candidate (partisanship, incumbency status, prior elective experience) and their electoral context (year, state, congressional district) is included for all observations. FEC candidate identification numbers are provided for all candidates with available identifiers. For convenience, we use this auxiliary information to merge our data with other relevant candidate and district-level information. Data on general election outcomes for pertinent candidates (i.e., primary election winners) are merged from the MIT Election Data and Science Lab^29^. Previous presidential vote share by congressional district is merged from The Downballot (https://www.the-downballot.com/p/the-downballots-calculations-of-presidential). State-level information on primary election participation rules is recorded from Open Primaries (https://openprimaries.org/rules-in-your-state/). Table 3 displays variable names, descriptions, and a sample observation from the dataset of biographical narratives.

**Table 3:** Columns from Example Entry in the CampaignView Biographical Narratives Dataset.

Variable	Description	Example
candidate_webname	Name of the candidate, standardized by year	Alma Adams
state_postal	Postal abbreviation for the candidate’s state	NC
cd	Congressional district for the candidate’s election	12
year	The year of the candidate’s election	2020
primary_type	The electoral rules governing the candidate’s primary election	Partially-Closed
dem_prez_vote	Democratic vote-share from the most recent presidential election	70.1
cand_party	Partisanship of the candidate	Democrat
inc	The candidate’s status as an incumbent member of the U.S. House	Incumbent
quality_cand	Candidate’s status as incumbent (2), prior office-holder (1), or amateur (0)	Experienced
win_primary	Whether (1) or not (0) the candidate won the primary election	1
primary_pct	Percent of the primary election vote-share garnered by the candidate	88.1
win_general	Whether (1) or not (0) the candidate won the general election	1
general_pct	Percent of the general election vote-share garnered by the candidate	100.0
biography_text	The biographical text featured on a candidate’s website	[text string]
housegov_bio	Whether (1) or not (0) the featured text is taken from a House.gov website	0
FECCandID	Identifier produced by the Federal Election Commission for candidates	H4NC12100
BioGuideID	Identifier from Congress.gov, which matches with legislative documentation.	A000009

### Policy Platforms

Each observation in this dataset is a platform point for an individual candidate’s campaign platform in a given year. The number of observations for a candidate in a given year varies depending on the number of platform points present in their campaign platform. This dataset’s main feature of interest is the policy text associated with each platform point. All observations for a candidate in a given year are assigned a sequential identifying variable; this can facilitate the aggregation of platform points into a single campaign platform while preserving their intended ordering. If a subheading accompanies the platform point, this associated text is stored in a separate text column. Each platform point is designated one of fourteen Major Policy Topic codes, outlined in Tables 1 and 2. Identical cleaning procedures are applied to these text data as described above; the same auxiliary information about each candidate and their electoral context is also provided. Table 4 displays variable names, descriptions, and a sample observation from the dataset of policy platforms.

**Table 4:** Columns from Example Entry in the CampaignView Policy Platforms Dataset.

Variable	Description	Example
candidate_webname	Name of the candidate, standardized by year	Alma Adams
state_postal	Postal abbreviation for the candidate’s state	NC
cd	Congressional district for the candidate’s election	12
year	The year of the candidate’s election	2020
primary_type	The electoral rules governing the candidate’s primary election	Partially-Closed
dem_prez_vote	Democratic vote-share from the most recent presidential election	70.1
cand_party	Partisanship of the candidate	Democrat
inc	The candidate’s status as an incumbent member of the U.S. House	Incumbent
quality_cand	Candidate’s status as incumbent (2), prior office-holder (1), or amateur (0)	Experienced
win_primary	Whether (1) or not (0) the candidate won the primary election	1
primary_pct	Percent of the primary election vote-share garnered by the candidate	88.1
win_general	Whether (1) or not (0) the candidate won the general election	1
general_pct	Percent of the general election vote-share garnered by the candidate	100.0
issue_header	The subheading text for a featured policy platform point	[text string]
issue_text	The policy text for a given platform position	[text string]
policy_code	The public policy topic code assigned to the text	Healthcare
statement_id	An ID variable for the sequential ordering of a candidate’s platform points	1
housegov_issue	Whether (1) or not (0) the featured text is taken from a House.gov website	1
FECCandID	Identifier produced by the Federal Election Commission for candidates	H4NC12100
BioGuideID	Identifier from Congress.gov, which matches with legislative documentation.	A000009

## Technical Validation

### Validation of candidate inclusion, website coverage

We validate our list of ballot-eligible congressional candidates by comparing our data to secondary sources documenting primary election outcomes (e.g., New York Times election reporting). We seek to identify candidates’ campaign websites through three separate collection efforts: searching twice before the candidate’s primary (one month prior and one week prior) using online search engines and repositories, and again after the election season via the Internet Archive’s Wayback Machine. Through this exhaustive data collection procedure, we make every effort to identify the universe of available campaign websites, but some candidates do not have an online campaign presence.

We were unable to identify a biographical narrative for 14.99% of the 6,016 major-party candidates in our data, a campaign platform for 25.08%, and a campaign website more broadly for 11.9%. In Table 5, we explore factors correlated with missing campaign websites, biographies, and platforms. We regress missingness in campaign website content on candidate covariates, such as partisanship and fundraising, and district covariates, such as primary election rules and contestation. Candidates who competed in contested primaries, raised substantial funds, and/or served as members of Congress were significantly less likely to have a missing campaign website, biographical narrative, or campaign platform. These patterns suggest a strong link between maintaining a campaign website and running a serious, competitive campaign. Indeed, three-quarters of candidates without a campaign website raised less than $100,000 for their congressional races. Among all general election winners, 88.3% had a campaign website that featured a campaign platform and 99.0% presented a campaign biography on that site.

**Table 5:** Predictors of missing campaign websites, biographies, and platform content. Logistic regression coefficients (standard errors in parentheses) indicate the relationship between missingness indicators and candidate-level covariates (e.g, incumbency status, party affiliation, fundraising) as well as district-level covariates (e.g., primary election type, contestation). Asterisks denote statistical significance at the 95% confidence level.

	*Dependent Variable: Missingness*		
	Website	Biography	Platform
No Incumbent in Election	0.029	0.148	− 0.113
	(0.103)	(0.094)	(0.083)
Primary Type: Open	0.054	0.069	0.188^*^
	(0.099)	(0.090)	(0.070)
Primary Type: Non-Partisan	0.033	0.115	0.155
	(0.139)	(0.125)	(0.098)
Unopposed Primary	− 0.342^*^	− 0.349^*^	− 0.320^*^
	(0.099)	(0.090)	(0.071)
Republican Candidate	− 0.029	0.054	0.238^*^
	(0.093)	(0.084)	(0.065)
Prior Office-Holder	− 0.385^*^	− 0.384^*^	0.291^*^
	(0.184)	(0.159)	(0.101)
Current Incumbent MC	− 2.967^*^	− 2.000^*^	− 0.314^*^
	(0.722)	(0.341)	(0.139)
Logged Fundraising	− 0.222^*^	− 0.194^*^	− 0.136^*^
	(0.009)	(0.008)	(0.006)
2020	0.280^*^	0.318^*^	0.120
	(0.109)	(0.102)	(0.079)
2022	− 0.286^*^	0.089	0.015
	(0.116)	(0.104)	(0.080)
Constant	− 0.298^*^	− 0.292^*^	− 0.009
	(0.140)	(0.129)	(0.105)
Observations	6,016	6,016	6,016
Log Likelihood	− 1,586.370	− 1,913.910	− 2,983.943
Akaike Inf. Crit.	3,194.741	3,849.821	5,989.885

### Validation of candidate name standardization

A key contribution of our database is its temporal coverage, encompassing multiple election cycles. In some cases, official candidate names from the Secretary of State or State Board of Elections vary across election cycles (e.g., Adam B. Schiff, Adam Schiff, or Schiff, Adam B.). To better facilitate cross-year candidate-level comparisons, we standardize the spelling and formatting of names across all elections observed in our data.

To implement this standardization, we first convert all candidate names to a consistent format: First [Middle] Last [Suffix]. Next, we determine whether a given candidate appears across multiple election years in our data. Specifically, we employ a probabilistic record linkage approach using pre-trained word embeddings^30^. This approach measures the semantic similarity of a pair of candidate names based on their proximity in embedding space. We conduct our fuzzy matching procedure on candidate names, with candidate party and state serving as blocking variables. We do not block on congressional district because our data span decennial redistricting, during which district boundaries—and thus district identifiers—frequently change. We review all linked records with an estimated match probability of less than 50% by hand and remove incorrect matches. When linking candidate names across the 2018 and 2020 elections, 15 matches were deemed incorrect. When linking candidate names across 2020 and 2022, only one matched case was deemed incorrect. No linkages were deemed incorrect when linking candidate names across 2018 and 2022. As a final validation step, we examine all linked candidates with inconsistent FEC identification numbers across election years. In all these cases, inconsistencies were attributable to a candidate running in a different congressional district across years, which generates a new FEC ID, rather than an incorrect matched case.

### Validation of hand-labeled topical codes

We validate the hand-labeling of Major Policy Topic codes assigned during data collection to each platform point in a candidate’s campaign platform. To do so, we tasked coders who collected text during the 2022 primary elections with re-labeling a random sample of 20% of the campaign platforms in our corpus (N=43,465), totaling to 8,584 platform points (i.e., documents). Coders were not assigned to relabel any campaign platforms they had labeled during their initial data collection effort. Percent agreement between re-labeled documents and their original topical coding was 80%. The Cohen’s Kappa statistic between coders, which computes the level of agreement while accounting for random chance, is 0.84; the weighted Cohen’s Kappa is 0.74. These statistics reflect an extremely high rate of agreement between raters. Upon manually reviewing statements for which there was inter-rater disagreement, we found that most disagreements came from statements labeled as “Unknown/Other.”

## Usage Notes

Recall that the units of analysis in our biographical narrative and policy platform datasets vary. Because of this, workflows for basic usage and aggregation will differ across these datasets. In our biographical data, each row is a candidate-year. If users seek to compare candidate self-presentation across time, they should uniquely identify candidates using the variables: candidate_webname, state_postal, and cand_party. We do not recommend using cd because many candidates ran in numerically different districts in our data due to redistricting. Be advised that only incumbents possess a BioGuideID, and some candidates lack a FECCandID because they never filed with the Federal Election Commission, so we do not recommend aggregating text using these identifying variables.

In our platform data, each row represents a candidate-year-platform point. Platforms can be aggregated at the candidate-year level by concatenating text and uniquely identifying candidates with the variables: candidate_webname, state_postal, cd, year, and cand_party. To maintain the order of platform points, rows should be sorted by these variables, along with statement_id. Multiple variables are required for unique identification because, in a few cases, several candidates sharing the same name ran in the same congressional district in the same year. Once again, we do not recommend aggregating text by FECCandID or BioGuideID. In the Harvard Dataverse, we provide code for aggregating individual platform points into complete platform documents at the candidate-year level. We also provide our datasets in this aggregated format.

**Fig. 3:**
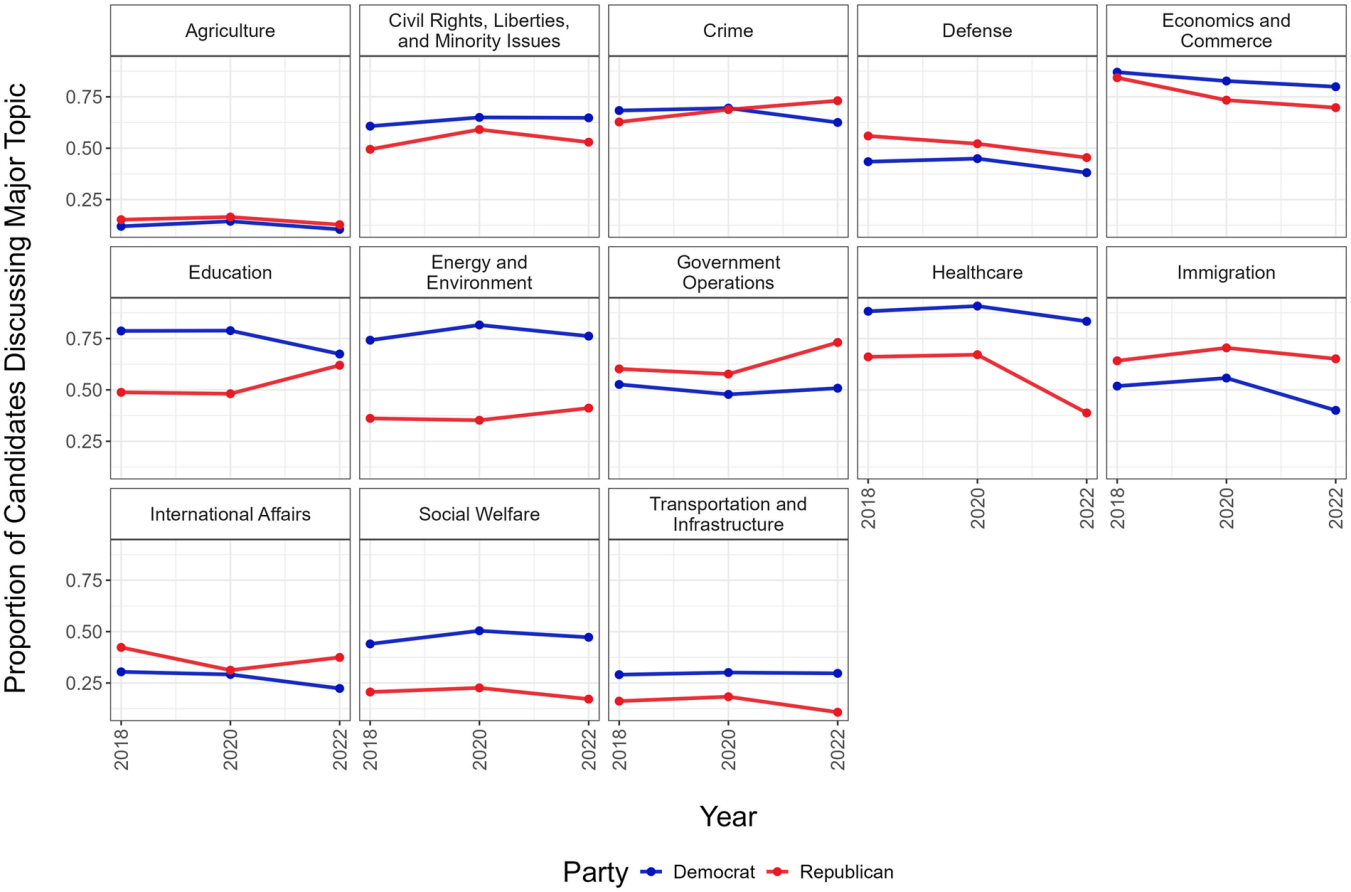
Partisan and Temporal Dynamics in Campaign Platform Major Topic Content. Proportion of candidates discussing each Major Policy Topic in their campaign platforms (y-axis) by election year (x-axis). Lines indicate party affiliation, showing trends in issue emphasis over the election cycles from 2018 to 2022.

CampaignView is well-suited to facilitate research on diverse questions, including partisan differences in policy agendas and the temporal dynamics of agenda setting. Figure 3 illustrates the proportion of candidates whose platforms address each of our Major Policy Topic codes, disaggregated by party and year. For certain topics, substantial partisan differences persist across years. Democrats consistently emphasize “Energy and Environment”, “Healthcare”, and “Social Welfare”, whereas Republicans discuss “Immigration” and “Government Operations” more frequently. Other topics exhibit notable temporal variation. Before 2022, Democrats were roughly 25 percentage points more likely than Republicans to discuss “Education”, a gap that narrowed significantly to about five percentage points in 2022. Trends in discussions of healthcare reveal a different pattern: in 2020, Democrats were 23 percentage points more likely to discuss “Healthcare” compared to Republicans, and by 2022, this difference widened to over 40 percentage points—driven by a marked decline in issue uptake among Republicans.

Users who are interested in working with campaign platform data related to a specific topic or policy area should keep several usage notes in mind. First, as we discussed above, our Major Policy Topic codes classify the topical content of the *majority* of the text in a given platform point. Candidates may discuss a single issue across multiple platform points, and this may go undocumented in our topical coding. For example, we classify discussions of Women’s Issues under the “Civil Rights, Liberties, and Minority Issues” MTC, but, oftentimes, related policy discussions also appear within text classified under the “Health” MTC (e.g., reproductive healthcare or breast cancer). Table 6 outlines other specific policy areas that may more often appear under multiple MTC classifications. Individuals who are interested in specific, rather than broad, issues or policies (e.g., abortion, Medicaid, No Child Left Behind) may have greater success identifying relevant text using a more tailored document discovery method. For a review of potential workflows, see King *et al*.^31^ or Case and Porter^32^.

**Table 6:** Codebook Crosswalk for Common Policy Topical Codes. footnoteThese usage notes illustrate common topical alignment and discrepancies between our Major Policy Topic codes, Policy Agendas Project (PAP) codes, and Congressional Research Service (CRS) policy areas. The table highlights instances where our coding diverges due to the distinct context of electoral campaign texts.

Major Policy Topic	Corresponding PAP Topic	Corresponding CRS Policy Area
Agriculture	Agriculture (Note: statements about trade may fall under the “Economics and Commerce” or “International Affairs” MPTs)	Agriculture and Food
Civil Rights, Liberties, and Minority Issues	Civil Rights; Social Welfare (Note: statements about abortion may fall under “Health” MPT; statements about age discrimination may fall under the “Social Welfare” MPT)	Civil Rights and Liberties, Minority Issues; Native Americans (Note: statements about abortion may fall under the “Health” MPT)
Crime	Law and Crime (Note: statements about child abuse may fall under the “Social Welfare” MPT)	Crime and Law Enforcement (Note: statements about terrorism may fall under the “International Affairs” MPT)
Defense	Defense (Note: statements about international alliances and foreign operations may fall under the “International Affairs” MPT)	Armed Forces and National Security (Note: statements about alliances and international affairs may fall under the “International Affairs” MPT)
Economics and Commerce	Macroeconomics; Domestic Commerce; Labor; Foreign trade (Note: statements about national budget may fall under the “Government Operations” MPT; statements about trade may fall under the “International Affairs” MPT)	Economics and Public Finance; Commerce; Finance and Financial Sector; Taxation; Foreign Trade and International Finance (Note: statements about the national budget may fall under the “Government Operations” MPT; statements about international trade may fall under the “International Affairs” MPT)
Education	Education	Education
Energy and Environment	Environment; Energy; Public Lands (Note: statements about Indigenous affairs may fall under the “Civil Rights and Liberties” MPT)	Energy; Environmental Protection; Public Lands and Natural Resources
Government Operations	Government Operations	Congress; Government Operations and Politics
Immigration	Immigration	Immigration
Healthcare	Health	Health
International Affairs	International Affairs (Note: statements about terrorism may fall under the “Crime” MPT)	International Affairs (Note: statements about trade may fall under the “Economics and Commerce” MPT)
Social Welfare	Social Welfare; Housing (Note: statements about Social Security may be included in the “Health” MPT when discussed in conjunction with Medicare/Medicaid; statements about child care may be included in the “Civil Rights, Liberties, and Minority Issues” MPT)	Housing and Community Development; Social Welfare
Transportation and Infrastructure	Transportation	Transportation and Public Works; Water Resources Development (Note: statements about water quality and environmental issues may appear under the “Energy and Environment” MPT)

While our coding framework broadly aligns with established coding schemes from the Policy Agendas Project (PAP) and the Congressional Research Service (CRS), we introduce several modifications due to the distinct nature of electoral campaign texts: policy areas that topically align in a legislative context do not always align in electoral position-taking. For instance, in campaign platforms, discussions of trade are commonly embedded within broader foreign policy statements, categorized under our “International Affairs” topic code, whereas the Policy Agendas Project categorizes these under “Economics & Commerce.” Table 6 comprehensively maps the relationships between our Major Policy Topic codes, Policy Agenda Project codes (PAP codes), and Congressional Research Service policy areas (CRS codes).

## Supplementary information

Supplementary Materials

## Data Availability

We used no customized or proprietary software for the creation of our databases. The replication code for cleaning text data is publicly available in the Harvard Dataverse.
