# Supplementary material for: CampaignView, a database of policy platforms and biographical narratives for congressional candidates
Source: Sci Data. 2025 Jul 15;12:1237. doi: 10.1038/s41597-025-05491-x (PMC12264109; doi:10.1038/s41597-025-05491-x)
Supplement: Supplementary file 1 — Supplementary Materials [file 41597_2025_5491_MOESM1_ESM.pdf]

CampaignView, a database of policy platforms and biographical narratives for congressional candidates

Supplementary Materials

Contents

A Coding Instructions for Human Annotators **1**

A Coding Instructions for Human Annotators

Codebook For Primary Elections Website Collection & Coding

Updated 02/27/2024

Candidate Information

Name: Select the name from the dropdown menu for each candidate on your list. If a candidate is not listed, please select "other" and type the candidate's name (and flag this candidate).

State and District: Enter the candidate's state and district. Both of these should be listed in the Google Sheet. You should also cross reference this with the state and district on the FEC website.

FEC ID: Go to https://www.fec.gov/. You can search by state and congressional district to view all candidates who have filed with the FEC. The incumbent candidate for the district will have a blue dot by their name. To get a candidate ID, please click on the candidate's name and copy and paste the ID directly.

Incumbent: If the candidate has a blue dot next to their name on the FEC website, they are an incumbent in Congress. You do not need to verify whether they are the current sitting incumbent for their specific district.

Biography or "About Me" Pages

These types of pages include biographical text about the candidate. Look on the menu of a candidate's webpage for any of the following. Select "yes" for this Qualtrics question if one of these pages exists.

- Bio or Biography
- About Me
- Meet the Candidate
- My Story
- Who is [Name of Candidate]?

FAQS:

What if there is no dedicated biography page?: Check to see if there is any descriptive information about the candidate on the website's homepage. If no text is available, select "No" for this Qualtrics question.

What if there is only a video on the bio page?: Check to see if there is any descriptive information about the candidate on the website's homepage. If no text is available, select "No" for this Qualtrics question.

What if there is text, but it is a picture (i.e., cannot be copied or pasted)?: If there is only certain text that is an image, leave this text out and proceed forward. If most of the text is an image, flag this candidate in the survey, add to the notes "biography text as an image, and check with a coding supervisor.

What if I see a button that says "Read More" on a candidate's biography?: Your goal is to collect all the text from a candidate's webpage. Please ensure you click the "read more" or "+" to reveal all text and copy/paste that into the Qualtrics form.

Example pages:

- https://chiproy.com/meet-chip/
- https://www.carl4congress.com/biography
- https://www.brucefornc.com/about

Candidate Race: The "Bio" or "About Me" page often references a candidate's race when they are not white. If a candidate is racially ambiguous, check endorsements or local media coverage for information on the candidate's race. Only if no other information is available, reference photos of the candidate.

Candidate Gender: The "Bio" or "About Me" page often references a candidate's gender identity through their pronoun usage. If a candidate's gender identity is ambiguous, check endorsements or local media coverage for information on the candidate's gender identity. Only if no other information is available, reference photos of the candidate. Use the "Other" if the candidate identifies as non-binary or uses plural pronouns exclusively.

Candidate Quality: We are interested in determining what *most qualifies* a candidate to hold elected office. Use the codes below to assign "candidate quality." In some cases, this will be their current occupation. In others, it may be something a candidate did previously. In general, for occupations code 13 and higher, select the highest coded position if a candidate has ever held any of these. For occupations code 12 and lower, please select the most recent and significant position.

If a candidate ever held a position they were elected to, please use that here.

30 = Current incumbent
29 = State-wide elected office (governor, sec. state, treasurer, etc.)
28 = State senator
27 = State representative
26 = Mayor
25 = Judge (elected)
24 = District or prosecuting attorney
23 = City council member, Selectman, Alderman
22 = Other elected office
21 = Previous incumbent in most recent Congress who lost
20 = Former House member (not most recent Congress)
19 = Former senator
18 = Attorney general
17 = State's attorney
16 = Commissioner
15 = Alderman
14 = Sheriff
13 = Treasurer
12 = Business/business owner
11 = Journalist
10 = Lawyer
9 = Judge (non-elected)
8 = Minister
7 = Farmer/rancher
6 = Military
5 = Local party leader/activist
4 = Doctor/Dentist/Vet/Nurse/PA
3 = Teacher/professor/educator
2 = Other government-non-elected/former congressional aide or advisor
0 = Indicates no electoral experience / political novice

FAQS:

What if the candidate has previously held any government office (13 or higher) but currently has another occupation?: Code the candidate's prior office.

What if the candidate has significant military experience but now works in another occupation 12 or lower?: If the current occupation is noteworthy (e.g., business CEO, doctor, lawyer), code the candidate's professional experience. If the current occupation is *not* noteworthy (e.g., would otherwise be assigned a "0"), code the candidate's military experience. In all cases, be sure to include the candidate's military experience in the subsequent question.

What if the candidate works for a business but is not necessarily the owner or in a leadership position?: Code that candidate as a "0." If a candidate identifies as an entrepreneur, code as a "12."

What if the candidate describes themselves as something (e.g., a small business owner, an activist, or an educator) but is somewhat vague about what that means? Code the candidate's self-described occupation that most qualifies them for office.

How does the candidate describe the occupation that most qualifies them for office? From the previous question, type out the *exact* occupation the candidate held that was used for the determination in the prior question. If no occupation exists, please type NA.

Has the candidate been elected to office previously?: For a quality score of 13 or higher, we want to ensure the candidate won elected office. Some positions are elected in certain states but appointed in others (e.g., Secretary of State). This can be verified through language in the bio that says "elected" or "appointed." If it is unclear from the biography, you can verify an election was held by searching Google for election results. If you cannot find any information, flag the candidate at the end of the survey and add a note.

Military Background: If the candidate has military experience, mark this as yes.

Legislative experience: If the candidate has held legislative office (i.e., Congress, a state senate or house, a general assembly) at any point, mark this as yes.

Platform or "Issues" Pages

These types of pages include information on. Look on the menu of a candidate's webpage for any of the following:

- Issues
- My Positions
- Platform

FAQ:

What if the issue page has only videos?: Select "No" for this Qualtrics question.

What if there is text, but it is a picture (i.e., cannot be copied or pasted)?: If there is only certain text that is an image, leave this text out and proceed forward. If a majority of the text is an image, flag this candidate in the survey, add to the notes "biography text as an image, and check with a coding supervisor.

What if there is text, but it is all one paragraph?: Select "Yes" for this Qualtrics question and add this text to the "lead-in" text field. Please also flag this candidate and add a note to the notes section at the end of the survey.

What if there is text but no sub-headings to copy/paste?: Copy and paste the body text into the Qualtrics form, select a main heading category, and leave the sub-heading category blank.

What if the sub-heading appears to be about a different topic than the text? For example, the sub-heading is "foreign policy," but the text is about immigration: Always label a topic's "main heading" based on *your* best judgment. While we want to try to make the main heading and sub-heading match to capture a candidate's intentions. Sometimes, a candidate will intentionally *mis*label a topic. For example, if a Democrat is running in a conservative district, she may label a topic about abortion "Protecting Your Rights" or "Healthcare Alternatives." In these cases, assigning a main heading best fits the text is best.

What if a platform point is about multiple things? Like Medicare and Social Security or Foreign Policy and Military?: Candidates often house multiple topics under one heading. If this occurs, pick the topic that best fits the text. If you are torn between issues, ask a coding supervisor.

What happens if I run out of room on my Qualtrics form?: If an issue exceeds 40 items (the maximum allotted on the Qualtrics form), submit your survey and start a second one. Copy and paste the candidate's name, select "No" for biography text, and make your 21st issue the first issue on the new survey. *Note that after 20 issue items, you must submit that page and go to the next.*

What if I see a "Read More" button for candidate issues?: Your goal is to collect all the text from a candidate's webpage. Please ensure you click the "read more"or "+" to reveal all text and copy/paste that into the Qualtrics form.

What if there are downloads about candidate issues?: Contact a coding supervisor.

Issue Examples by Category

[Examples match those outlined in Table 1]
